# How do the severe acute respiratory coronavirus 2 (SARS-CoV-2) and its variants escape the host protective immunity and mediate pathogenesis?

**DOI:** 10.1186/s42269-022-00945-3

**Published:** 2022-10-12

**Authors:** Rashed Noor

**Affiliations:** Department of Life Sciences (DLS), School of Environment and Life Sciences (SELS), Independent University, Bangladesh (IUB), Plot 16, Block B, Aftabuddin Ahmed Road, Bashundhara, Dhaka 1229 Bangladesh

**Keywords:** COVID-19 pandemic, Severe acute respiratory syndrome β coronavirus 2 (SARS-CoV-2), SARS-CoV-2 variants, Host protective immunity, Avoidance of host innate immunity, Vaccines

## Abstract

**Background:**

To protect the global population from the ongoing COVID-19 pandemic caused by the severe acute respiratory β-coronavirus 2 (SARS-CoV-2), a number of vaccines are currently being used in three dosages (i.e., along with the booster dose) to induce the immunity required to combat the SARS-CoV-2 and its variants. So far, several antivirals and the commercial vaccines have been found to evoke the required humoral and cellular immunity within a huge population around world. However, an important aspect to consider is the avoidance mechanism of the host protective immunity by SARS-CoV-2 variants.

**Main body of the abstract:**

Indeed, such an immune escape strategy has been noticed previously in case of SARS-CoV-1 and the Middle East Respiratory Syndrome coronavirus (MERS-CoV). Regarding the SARS-CoV-2 variants, the most important aspect on vaccine development is to determine whether the vaccine is actually capable to elicit the immune response or not, especially the viral spike (S) protein.

**Short conclusion:**

Present review thus focused on such elicitation of immunity as well as pondered to the avoidance of host immunity by the SARS-CoV-2 Wuhan strain and its variants.

## Background

Since the middle of March 2020, the serious health threat of this century so far has been caused by the severe acute respiratory syndrome γ coronavirus 2 (SARS-CoV-2), a ~ 30 kb RNA virus, which is also known as the 2019-nCoV coronavirus, a member of *Nidovirales* order of the family *Flaviviridae*, resulting in COVID-19 pandemic with 6,325,785 deaths out of 540,923,532 confirmed cases of COVID-19 so far (WHO 2022; Noor 2021a). As of 20 June 2022, a total of 11,912,594,538 vaccine doses have been administered (WHO 2022). SARS-CoV-2 originated from Wuhan, Hubei province of China in the last of December in 2019, and then spread around the world within three months as a consequence of the travel-related infection together with the concomitant second wave of infection currently especially in Europe (Cacciapaglia et al. 2020; Haider et al. 2020). Prior to this COVID-19 pandemic, the Middle East Respiratory Syndrome Coronavirus (MERS-CoV) disease outbreak in Saudi Arabia in 2012; and the acute respiratory syndrome (SARS) epidemic in 2003 was significant (Zhang et al. 2020).

Historical similarity with the present COVID-19 pandemic lies with the Hong Kong influenza in 1968, Asian influenza in 1957–1958, Spanish influenza in 1918–1920, and the Russian influenza in 1889–1892 (Cacciapaglia et al. 2020; Haider et al. 2020; Zhang et al. 2020; Noor and Maniha 2020). The drastic transmission of SARS-CoV-2 during the second wave starting from March 2021, the mutation dynamics of the virus extensively being analyzed which projected a hotspot mutation at the position of D614m generating the G614 variant, achieving the replacement of glycine in its receptor binding domain (RBD) of the viral spike (S) protein, causing the trouble in the vaccine-mediated induction of the host immune response (Noor 2022a, 2022b; Korber 2020; Zhang et al. 2020). Eventually, the genomic variations arose from the wild-type strain of SARS-CoV-2 strains; and several variants of concern (VOCs) and the variants of interest (VOI) emerged (Noor 2022a; Otto et al. 2021). The SARS-CoV-2 variants, i.e., Alpha (B.1.1.7), Beta (B.1.351), Gamma (P.1) and Delta (B.1.617.2) evolved in late 2020; while the Omicron variant (B.1.1.529) emerged in November 2021 (Boehm et al. 2021; Noor 2022a, 2022b). The constant mutations within the S protein raised questions about the efficacy vaccine efficiency which was further resolved by the use of the booster dose of vaccines (Chenchula et al. 2022; Noor 2022a, 2022b).

Scientists around the world tried their best to resolve the genetic- and the immunological issues related to the COVID-19 pathogenesis as well as for the development of the therapeutic strategies including the development of antiviral drugs (like remdesivir, ribavirin, favipiravir, hydroxychloroquine, lopinavir, ritonavir, arbidol, bamlanivimab and ostalmovir); the soluble angiotensin-converting enzyme 2 (ACE2) or the serine protease inhibitor camostat; and vaccines including ChAdOx1 nCoV-19 (Oxford/ AstraZeneca), Pfizer-BioNTech (BNT162b1), Moderna (mRNA 1273), BBIBP-CorV (Sinopharm), Sputnik V/ Gam-Covid-Vac (Gamaleya) vaccines against SARS-CoV-2 and its variants (Noor 2020, 2021b, 2022b; Samantaray et al. 2021).

However, the escape of the host protective immunity by these strains with the concomitant production of the mutant/ variants, i.e., mutations in the viral spike (S) protein have made the virus incrementally lethal to mass public (Mattoo et al. 2022; Noor et al. 2022; Awadasseid et al. 2021; Dos 2021; Hoffmann et al. 2021; Kikkert 2020; Wrapp et al. 2020). For example, the dreadfulness of the variants can be interpreted by the B.1.617 lineage variant in India which may get entry into lung and intestine cells, exhibited the neutralizing antibody evasion strategy; stimulating a sharp rise in the viral count; and couldn’t be hindered by applying the antiviral drug bamlanivimab neither with the Pfizer-BioNTech BNT162b2 vaccine; resulting in the rapid spread of this variant (Hoffmann et al. 2021). In contrast to the wild-type SARS-CoV-2 Wuhan strain, the variants showed higher transmissibility with potent virulence and antigenicity as discussed by several groups (Table 1). Present review therefore highlighted the possible impact of the viral variants on the instigation of the appropriate immunity provoked by the vaccines.

**Table 1:** Comparison regarding the change in transmissibility, virulence and antigenicity in the wild-type SARS-CoV-2 and its emerging variants

SARS-CoV-2 and its variants	Transmissibility	Virulence	Key mutations influencing antigenicity	References
Wild-type SARS-CoV-2	First originated in Wuhan, China	Binding of receptor binding domain (RBD) of the spike (S) protein to the human angiotensin converting enzyme 2 (hACE2), mediating the viral entry	None	Noor et al. (2022)
Alpha variant (of B.1.1.7 lineage)	Originated in the UK. Transmissibility increased by 29% (160 countries) as of June 2021	The emerging variants of the Wuhan strain of SARS‐CoV‐2 possess the key mutations, especially in the RBD of the spike (S) protein that interacts with (hACE2 may instigate significant alterations in the interaction between SARS-CoV-2 and the host. Such mutations may accelerate the mechanism of RBD binding to the hACE-2 of the S protein, enhances glycosylation of this S protein at the antigenic sites, which in turn, results in the proteolytic cleavage of the S protein with concomitant entry into the host cells. New VOCs may take their entry even more easily imparting increased viral replication frequency/ viral shedding, resulting in more lethality with serious tissue impairment as well as hyper inflammation. Enhancement of viral replication and evasion of the neutralizing antibodies are another strategies of SARS-CoV-2 pathogenesis	Total 23 mutations, Key mutations: H69-V70del, N501Y, and P681H, conferring viral entry	Noor et al. (2022) Campbell et al. (2021), Kumar et al. (2022)
Beta variant (B.1.351 lineage)	Originated in South Africa. Transmissibility increased by 25% (113 countries) as of June 2021		Key mutation: N501Y within the RBD domain of S protein, conferring n RBD high affinity to bind hACE-2	Noor et al. (2022), Campbell et al. (2021), Kumar et al. (2022)
Gamma variant (of P.1 lineage)	Originated in Brazil. Transmissibility increased by 38% (64 countries) as of June 2021		Total 17 mutations, conferring viral entry	Noor et al. (2022), Campbell et al. (2021), Kumar et al. (2022)
Delta variant (B.1.617.2 lineage)	Originated in India. Transmissibility increased by 97% (62 countries) as of June 2021		Key mutations: E484Q, L452R, P681R, conferring viral entry. L452R also facilitates viral entry and antibody evasion.	Noor et al. (2022), Campbell et al. (2021), Kumar et al. (2022)
Omicron variant (B.1.1.529 lineage). The Omicron variant has mutated into three lineages: BA.1, BA.2, and BA.3, as of February 2022	Originated in South Africa. This variant of concern (VOC) had spread across 105 countries as of January 10, 2021		More than 50 mutations (Thirty mutations in spike (S) protein, alteration of 9 amino acids), conferring viral entry; and the evasion of neutralizing antibody	Noor et al. (2022), Campbell et al. (2021), Sanyaolu et al. (2022)

## Main text

### General scheme of SARS-CoV-2 pathogenesis

**Fig. 1:**
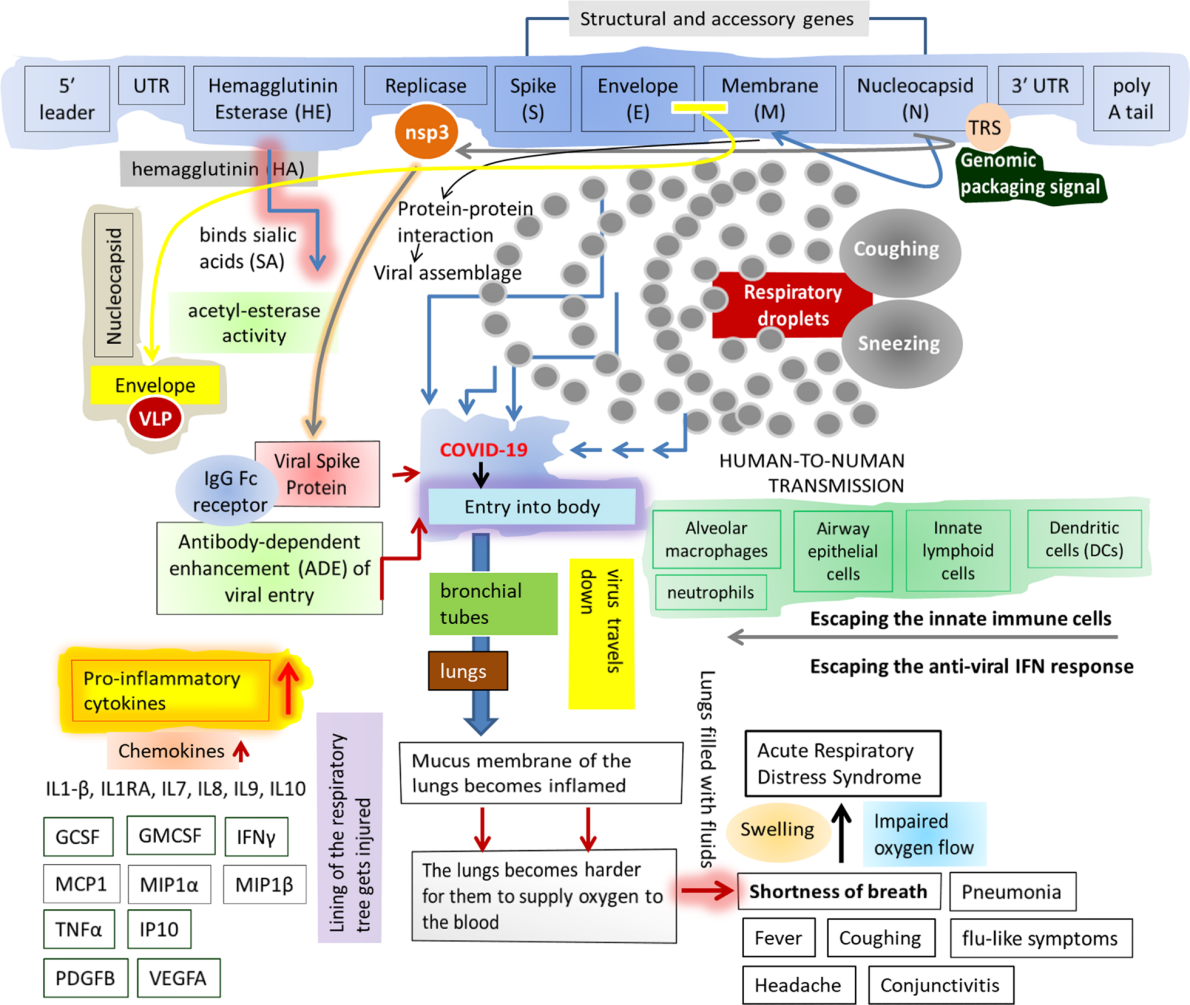
The RNA genome organization of SARS-CoV-2 is shown to decipher the entry and pathogenesis of the virus. SARS-CoV-2 enters the nasopharyngeal tract through the respiratory droplets ejected from coughing or sneezing by another person(s) in close contact. As shown, the hemagglutinin-esterase (HE) (1) acts as a hemagglutinin (HA), (2) it binds sialic acids (SA) on the surface glycoproteins, and (3) contains acetyl-esterase activity triggering the viral spike (S) protein-mediated host cell entry and subsequent proliferation across the mucosa. The nucleocapsid (N) protein works on two specific RNA substrates: (1) the transcriptional regulatory sequences (TRSs) and (2) the genomic packaging signal. Once inside the host membrane, the virus travels down bronchial tubes to the lungs; and as a result, the lining of the respiratory tree becomes injured which in turn irritates the nerves of the lining of the airway; and the resultant impact is the inflammation and hardening of the mucus membrane of the lungs making difficult to supply oxygen to the blood instigating the increased shortness of breath. The significant increase in the pro-inflammatory cytokines and chemokines (including IL2, IL7, IL10, GCSF, IP10, MCP1, MIP1α, and TNFα), i.e., the cytokine storm has been shown to induce the local inflammation generating the severe onset of the disease

SARS-CoV-2 enters the nasopharyngeal tract from the respiratory droplets following its movement across the bronchial tubes to the lungs, which in turn makes the mucous membrane of the lungs inflamed and hard which results in difficulty in supplying oxygen to the blood and hence the shortness of breath in combination with the acute cardiac injury, and severe pneumonia (Fig. 1) (Noor 2021a; Kikkert 2020). Indeed, the viral entry is facilitated by the receptor binding domain (RBD) within the viral spike (S) protein and the host angiotensin-converting enzyme 2 (ACE 2) receptor which in turn triggers both the host innate and adaptive immunity (Jackson et al. 2022; Noor 2021a, 2021b). The orchestrated protective network between the host airway epithelial cells, neutrophils, alveolar macrophages, dendritic cells (DCs), lymphocytes, toll-like receptors (TLRs), and the pathogen-associated molecular patterns (PAMPs) instigated by the pattern recognition receptors (PRRs) generate an anti-viral state within the lungs (Madden and Diamond 2022). However, the viral entry provokes the rush of the pro-inflammatory cytokines and chemokines; and such condition is already well known as the cytokine storm as shown in Fig. 1. Such impulsive response of the innate immune system may attack the host (self) protective system which in turn provokes the commencement of the acute respiratory distress syndrome (ARDS) together with major organ (most commonly kidney and liver) malfunction (Birhanu et al. 2022; Noor 2021a). Moreover, as a countermeasure against the innate defense machineries, i.e., the alveolar macrophages, airway epithelial cells, innate lymphoid cells, and DCs as stated above, the invading viruses may evolve activities suppressing such mechanisms which enhances the viral replication (V'kovski et al. 2021; Noor 2021a; Kikkert 2020). The equilibrium between the effectiveness of the innate and adaptive responses of the host and the capacity of virus to escape the host's immune responses thus dictates the disease syndrome (Gu et al. 2022; Kikkert 2020).

### SARS-CoV-2 genome in relation to its life cycle and pathogenesis

SARS-CoV-2, the SARS-CoV-1 (causing the epidemic in 2002) and the Middle East respiratory syndrome coronavirus (MERS-CoV) that generated in 2012, possess highly conserved genomic organization, with a large replicase gene which encodes the non-structural proteins (Nsps) (Abdelghany et al. 2021; Noor 2021a). The SARS-CoV-2 genomic RNA consists of 14 open reading frames (ORFs) of which the first two ORFs (pp1a/ab) encode polyprotein which are involved in the viral replication; and 16 non-structural proteins which are needed for the viral RNA transcription and replication of the virus trailed by the structural proteins (Noor 2020). Indeed, four major genes encoding ACE2 (the corresponding gene is located at Xp22.2 in host chromosome), IL-2, 7 and 10 (chromosomal locations: 4q27, 8q21.13 and 1q32.1, consecutively), TNF (gene location at 6p21.33), and VEGF (chromosomal location: 6p21.1) are associated with the development of the respiratory problems including the aspirin-sensitive respiratory disease (ASRD) (Veerabathiran et al. 2021).

As shown in Fig. 1, the SARS-CoV-2 RNA consists of a 5′ leader sequence followed by the untranslated region (UTR, containing the multiple stem loop structures needed for RNA replication and transcription) with the downstream genes encoding the hemagglutinin (HA)-esterase (HE), the replicase–transcriptase polyprotein, the spike protein (S, mediating attachment to the host receptor), envelope (E), facilitating the assembly and release of the virus), membrane (M, which is thought to give the virion its shape), nucleocapsid (N, triggering a structural change enhancing the affinity for viral versus nonviral RNA), and the 3′ UTR region (required for replication and synthesis of viral RNA) ending with poly adenine (A) tail (Bhat et al. 2021; Noor 2021a). N protein also binds to the nsp3 (encoding the papain-like proteases, PLpro) of the replicase complex and to the M protein, which in turn facilitates tethering the viral genome to the replicase–transcriptase complex (RTC) followed by the packaging of the encapsidated genome into new viral particles. The accessory genes (some of the products are essential for the viral pathogenesis) remain interspersed within the structural genes at the 3′ end flanking ORFs (Noor 2021a; Redondo et al. 2021).

Thus, the replicase is located at the upstream region of the (1) structural genes encoding spike protein, envelope membrane, nucleocapsid, and (2) the accessory genes (located with flanking ORFs at the 3ʹ terminus) which have been noticed to be essential for viral pathogenesis by inhibiting the host immune response (Beyer and Forero 2022; Noor 2021a, 2021b; Kikkert 2020). Based on such genome organization with their successive expression, the viral life cycle that is actually associated with the viral pathogenesis, involves the events of (1) the attachment and entry into the host followed by replicase protein expression, (2) the replication of the viral particles, and (3) viral RNA transcription trailed by the assembly of the viral components; and (4) ultimately the release of the mature viruses which reign pathogenic regime (Noor 2021a; Kikkert 2020). The replication of SARS-CoV-2 genome, involving viral and host proteins (for example, the DDX helicases) to accomplish RNA polymerization, proofreading and final capping within the infected cells appears as the central basis of the viral life cycle (Trougakos et al. 2021; Noor 2020; Kikkert 2020).

### Viral attachment and entry

The spike (S) glycoprotein on the surface of the SARS-CoV-2 is recognized principally by the host ACE2 receptors (in some cases by CD147 as well); and after attachment of the viral particle to the host cell, the S protein is cleaved by the human transmembrane protease serine 2 (TMPRSS2) so that the virus may enter the host cells by endocytosis or by direct fusion of its envelope with the host cell membrane (Noor 2021a, 2020; Kikkert 2020; Romano et al. 2020). Indeed, the attachment of the virus particle to the host cell is mediated by the interaction between the S1 region of the S protein (at the sites of RBD) and its receptor. After binding to the receptor, the virus enters the host cell cytosol by the acid-dependent proteolytic cleavage of the S protein usually by a cathepsin, followed by the fusion of the viral and cellular membranes mostly within acidified endosomes, and to some extent at the plasma membrane (Noor 2021a, b; Noor 2020; Kikkert 2020).

Next the translation of the replicase gene occurs into two co-terminal polyproteins, pp1a and pp1ab which are known to facilitate the viral RNA replication as stated previously (Romano et al. 2020; Wang et al. 2020). Specifically, the largest polyprotein PP1ab embeds all the non-structural proteins (Nsp1-16) and thereby form the complex replicase machinery which includes enzyme activities required for viral RNA transcription and translation (Romano et al. 2020). This is also to be noted that the hemagglutinin-esterase (HE) possesses the acetyl-esterase activity, acting as a hemagglutinin, has been reported to bind sialic acids on surface glycoproteins which in turn may facilitate the S protein-mediated viral entry and spread along the lung mucosa and hence triggers the viral pathogenesis (Wang et al. 2020).

### Viral RNA synthesis: replication and transcription

After the virus gets entry into the host cell, the infecting RNA serves as the messenger RNA (mRNA) which undergoes translation by host ribosomes to synthesize the viral replicative enzymes to produce fresh RNA genomes together with the mRNAs for the synthesis of the viral assembly machineries (Romano et al. 2020). Indeed, the SARS-CoV-2 RNA replication machinery is associated in a replication transcription enzyme complex anchored to host membranes generated from the endoplasmic reticulum (ER), and constitutes a range of functional proteins specifically the PP1ab, comprising the essential RNA-dependent RNA polymerase (RdRp, Nsp12), the zinc-binding helicase (HEL, Nsp13), enzymes required for mRNA capping (Nsp14, Nsp16), RNA proofreading (Nsp14), and the regulators of these proteins (Nsps 7–10) (Romano et al. 2020; Wang et al. 2020). In the replicase complex there are several other non-structural proteins (Nsps 3, 4 and 6) within the coronavirus which assemble into the replicase–transcriptase complex (RTC), consisting of spatially distributed and convoluted membranes (CVs) and double-membrane vesicles (DMVs) evolving from the endoplasmic reticulum (ER), for RNA synthesis and the transcription of the sub-genomic RNAs (Noor 2021a; Romano et al. 2020; Wang et al. 2020). A *cis*-regulatory element encoded within the SARS-CoV-2 RNA known as the packaging signal (PS), instigates the packaging of the viral genome into the ribonucleocapsid (Romano et al. 2020; Wang et al. 2020; Artika et al. 2020).

This is noteworthy that the CVs (where viral replication machineries anchor) and DMVs (the main site of viral RNA replication and transcription) are well protective environment for the viral genome so that SARS-CoV-2 becomes able (possibly by the uridylate-specific endoribonuclease activity imparted by Nsp15) to circumvent the detection of the dsRNA by the host innate immunity sensors, and thus the viral RNA is protected from degradation (Romano et al. 2020). The nsps also contain other enzyme domains and functions, including those important for the viral RNA replication (Noor 2021a; Raj 2020). Viral RNA synthesis produces both genomic and sub-genomic RNAs; and the later ones serve as mRNAs for the structural and accessory genes which may hinder the host immunity as stated earlier. Moreover, this is worth to note that the N protein may instigate the SARS-CoV-2 replication cycle as well as this protein is essentially involved in viral assembly and provoking the host response toward the viral infection (Noor 2021a, b; Romano et al. 2020; Wang et al. 2020; Artika et al. 2020).

### Protein synthesis, viral assembly and release of mature particles

Following replication and sub-genomic RNA synthesis, the viral structural proteins, S, E, M and N are translated; and with the aid of the intracellular trafficking signals (principally by the M and E proteins; and in some cases, the S proteins), these proteins are targeted and inserted into the ER–Golgi intermediate compartment (ERGIC) where the final assembly of virion components takes place (Kaushal and Noor 2022; Noor 2021a). The M proteins have been reported to direct most protein–protein interactions required for assembly of coronaviruses (Romano et al. 2020; Wang et al. 2020; Artika et al. 2020).

Besides, the nucleocapsid (N) phosphoprotein has also been demonstrated to play an imperative role during viral self-assembly as well as in the formation of the ribonucleoprotein (RNP); and the interaction between two N proteins are also necessary for the viral components’ assembly (Artika et al. 2020). Interestingly, the combined expression of both the M protein and E protein triggers the formation of virus like particles (VLPs). Eventually, the ability of the S protein to traffic to the ERGIC and interact with the M protein is critical for its incorporation into the virus particles. Finally, the M protein, the most plentiful structural protein, binds to the nucleocapsid as well as the PS (probably with the help the structural protein E) which completes the assembly process; and the virus particles are transported to the cell surface in vesicles following their subsequent release by exocytosis (Noor 2021a; Romano et al. 2020; Wang et al. 2020).

### SARS-CoV-2 variants and their increased transmissibility along with the capacity to hinder the host immune system induction by vaccines

The spike (S) protein has already been known to conduct the receptor binding and fusion of the viral and cellular membrane during the viral entry into the host cell; and the S1 domain within the RBD of the spike facilitates the ACE2 receptor binding whereas the S2 subunit remains engaged in the membrane fusion (Han et al. 2022; Noor 2021a, 2020). The study of several sequences of SARS-CoV-2 isolates unraveled the events of genetic variations within some genomic regions of the virus; especially, with the aspartic acid (S^D614^) and glycine (S^G614^) at residue 614 in the S protein (Zhang et al. 2020; Korber 2020). This is to be noted that the S1/S2 junction is further processed by a furin-like proprotein convertase; and both of these subunits are processed in the host within the S2 domain that is needed for the viral propagation (Korber 2020).

Indeed, as shown in Table 1, the events of antigenic draft producing the accumulations of mutations within the influenza viruses have been reported earlier (especially in the common cold coronaviruses OC43 and 229E as well as in SARS-CoV whereby a single amino acid change, the spike D480A/G in the RBD was noticed) and point mutations within the MERS-CoV; and it is to be noted that such drafting and reassortment confer the viruses the ability to resist the host protective immunity (including the antibodies which are supposed to neutralize the virus) and hence accelerating the viral transmissibility (Zhang et al. 2020; Noor and Maniha 2020; Korber 2020). Analyzing the SARS-CoV-2 spike protein evolution (i.e., identifying the spike amino acid variants) within a broad range of geographical locations using the bioinformatic tools and the Global Initiative for Sharing All Influenza Data (GISAID) SARS-CoV-2 sequence database during the ongoing COVID-19 pandemic facilitates the study of the possible mutations which in turn may bring innovations in the development of the current vaccination strategies (Noor et al. 2022; Khare et al. 2021; Korber 2020). Indeed, after the commencement of COVID-19 pandemic in the last of December, 2019, a new variant was noticed with a single D614G mutation in the spike (S) protein of SARS-CoV-2, spreading in Europe in early February; and surprisingly, the G614 was the dominant variant accompanied with dreadful infectivity (Noor et al. 2022; Korber 2020). However, according to the report of Zhang et al. (2020), the G614 genotype (mutation in the glycine residue) was noticed at low frequency (26%) in March 2020, which had a high accelerated transmissibility (up to 70%) by May 2020 (Zhang et al. 2020). This was comparable with the D614 variant having the mutation within aspartic acid residue since the SARS-CoV-2 with S^G614^ was noticed to be stabler and to infect the ACE2-expressing cells more proficiently than those with S^D614^ (Zhang et al. 2020).

This is to be noted that B.1.1.7 lineage that emerged in the UK, is now spreading across many geographic locations with a tremendously increased transmissibility compared with previously circulating strains (Hoffmann et al. 2021). Such an increased transmissibility may be associated to the N501Y mutation in the RBD of the S protein (Hoffmann et al. 2021; Tian et al. 2021). As stated earlier, the RBD in subunit 1 binds to the host ACE2 receptor, followed by activation of the spike by the transmembrane protease serine 2 (TMPRSS2) so that the subunit 2 (S2) may facilitate the fusion of the virus and the host cell membrane, facilitating the delivery of the viral RNA into the host.

Such a mechanism is important for the viral infection; and on the contrary, this is also important for the efficient vaccination as the spike protein serves as the primary target of monoclonal antibody therapies and the neutralizing antibodies generated through the administrations of vaccines. Like the B.1.1.7 variant, the B.1.351 (in South Africa) and the P.1 variant (in Brazil) have been noticed to harbor the spike RBD mutation E484K, which also reduced the neutralization capacity by the vaccine induced antibodies (i.e., the antibody evasion strategy), and thus those variants spread out with morbid effects (Hoffmann et al. 2021). This is to note the steep escalation of the COVID-19 cases in India within the last one month has been instigated by the B.1.617 variant harboring eight mutations within the S protein among which the RBD mutations L452R and E484Q modulate antibody-mediated neutralization (Hoffmann et al. 2021). Hence, any mutation in the spike protein may render the vaccine non-functional due to the target conformational change or displacement; and may alter the significant properties such as the efficiency of host cell entry as well as the susceptibility of the variants to the prescribed COVID-19 drugs (Noor et al. 2022b; Hoffmann et al. 2021).

### Immunopathology caused by SARS-CoV-2 and its variants

Upon entry of the virus into the respiratory tract, a prearranged cellular innate immune sensor recognizes it (Fig. 1), and a set of protective immune cells including the airway epithelial cells, the alveolar macrophages, the innate lymphocytes, DCs, TLRs-3, 7 and 8 are triggered to launch the typical anti-viral status along the lungs (Kaushal and Noor 2022; Kikkert 2020). Additionally, as stated earlier, the innate immune cells of the host are induced following the detection of PAMPs by the PRRs (Kikkert 2020). Indeed, the onset of COVID-19 results in the elevated leukocytes and the pro-inflammatory cytokines (these inflammatory indicators refer to the so-called cytokine storm in the patients with severe infection) including interleukins (ILs): IL2, IL7-10, IL1-β, interleukin-1 receptor antagonist (IL1RA); granulocyte colony-stimulating factor (G-CSF), Granulocyte–macrophage colony-stimulating factor (GMCSF), interferon (IFN)-γ, IFNγ-induced protein 10 (IP10), monocyte chemotactic protein-1 (MCP1), macrophage inflammatory protein 1 α (MIP1α), MIP1β, platelet derived growth factor(PDGF), vascular endothelial growth factor A (VEGFA), and the tumor necrosis factor (TNFα), all of which actually account for the severity of the disease syndrome (Bonnet et al. 2021; Noor 2021a, 2021b; Kikkert 2020; Noor 2020).

The connection of declining clinical state with the lessening viral counts and the commencement of a high immunological response (i.e., the elevated leukocytes), plus the presence of markedly elevated pro-inflammatory cytokines may intimate that the stern lung impairment is on the whole immunopathological event (Bonnet et al. 2021; Hembram 2021; Kikkert 2020). Indeed, besides such immune-mediated mechanisms, SARS-CoV-2 has also been noticed to influence the host cells by its cytocidal activity as observed through the cytopathic effects in the kidney cells as well as the formation of syncytia in lung tissues during viral replication. Moreover, the SARS-CoV-2 pathogenesis may involve the adaptive immune system whereby (1) T cells and the cytokines impart the disease progression potential; and (2) the humoral antibodies like IgG and IgM also play significant roles as can be seen from the event the antibody-dependent entry (ADE) of the SARS-CoV-2 into the host (Noor 2021a, 2020; Kikkert 2020; Li et al. 2020; Naqvi et al. 2020).

### Avoidance of the host protective immunity by SARS-CoV-2 and its variants

SARS-CoV-2 has been reported to act as interferon antagonists (the IFN-stimulated genes, ISGs may impart protective effects to host), to interfere with the PRR signaling such as TLRs, and to generate the non-productive inflammation, which in turn results in the cytokine storm as well as the viral shedding along the major organs because of the viral potential to avoid the host anti-viral interferon response (type I/III IFNs for the host defense) (Hembram 2021; Taefehshokr et al. 2020; Vabret et al. 2020). Especially, the increased levels of the pro-inflammatory IL-2, IL-7, G-CSF, MCP-1, MIP-1α, IP-10, and TNF-α has been shown to trigger the influx of neutrophils and other myeloid cells along the lung tissue, evoking the severe local inflammatory response (Kikkert 2020; Taefehshokr et al. 2020; Vabret et al. 2020).

Indeed, as shown in Table 2, the COVID-19 pandemic may be seriously escalated when the host immunity is significantly impaired, i.e., avoidance of the host immune sensors by SARS-CoV-2 during the viral life cycle, induction of cytokine storm in the infected individual, damage of the host interferon responses, suppression of the antigen presentation both by the major histocompatibility complex (MHC) class I and II, etc. (Noor 2021a, 2021b; Vabret et al. 2020). Hence the events within both the host immune responses against SARS-CoV-2 and the viral strategies to escape the host immunity are important to study which in turn may improve the existing knowledge on the viral pathogenesis as well as to determine the drug targets (Kikkert 2020; Taefehshokr et al. 2020; Vabret et al. 2020; Wan et al. 2020).

**Table 2:** A summary of host protective immune evasion strategies by SARS-CoV-2 and its variants

Immune evasion strategies	Mechanisms of viral escape of host immunity	References
Defective recognition of SARS-CoV-2 by the host	Loss-of-function mutations in the immune sensor, the toll like receptor gene *TLR7*, encoding TLR7 which acts as the pattern recognition receptor (PRR) to recognize the pathogen-associated molecular pattern (PAMP). Thus the initial PAMP-PRR interaction	Martin-Sancho et al. (2021), van der Made et al. (2020), Wan et al. (2020)
Avoidance of innate immunity during viral entry	A defective interferon (IFN) response to SARS-CoV-2 by host that is resulted due to the impairment of expression of the IFN-stimulated genes (ISGs) encoding mainly *LY6E*, whose product stops the viral replication onward	Martin-Sancho et al. (2021), Kikkert (2020), Snijder et al. (2020)
	Downregulation of several ISGs which specifically interferes the entry of SARS-CoV-2 spike (S) protein	
	Suppression of IFN-1 induced anti-viral state triggers hyper-inflammation and COVID-19 severity	Wan et al. (2020), Snijder et al. (2020)
	Defective endosomal factors which are actually directed to inhibit the entry of SARS-CoV-2	
Loss of control to inhibit SARS-CoV-2 replication	Loss of expression of the required RNA binding proteins which are supposed to hinder the viral RNA synthesis	Martin-Sancho et al. (2021), V'kovski et al. (2021)
	Lack of production of the cluster of endoplasmic reticulum (ER)/Golgi-resident anti-viral ISGs which are dedicated to suppress the genes required for viral assembly	
Curved membrane vesicles	Such modification of intracellular membranes makes the SARS-CoV-2 RNA replication easier	Klein et al. (2020)
Cap-snatching process	The host capping enzymes may be employed by SARS-CoV-2, resulting in viral mRNAs consisting of both the host capped small RNA (addition of a 7-methyl guanosine; and lacking of the 2′-O-methylation) and the virus-encoded RNA. Thus, the SARS-CoV-2 RNAs may escape recognition by the host innate immune RNA sensors	Beyer and Forero (2022), Mandilara et al. (2021), Kikkert (2020), Dai et al. (2020)
Avoidance of recognition by the melanoma differentiation-associated protein (MDA5) sensor	Avoid recognition by the MDA5 sensor which controls the innate immune response to SARS-CoV-2 in the lung epithelial cells. Viral endoribonuclease activity encoded in one of the non-structural genes may also hinder the recognition by MDA5 sensor	Yin et al. (2021), Kikkert (2020)
Evading host innate immunity by the viral endoribonuclease	Avoidance of the MDA5 recognition (as stated above)	Kikkert (2020), Drappier et al. (2015), Kindler et al. (2017)
	Avoidance of the protein kinase R (PKR), and the 2’-5’ Oligoadenylate Synthetase (the OAS/RNAse L system, which triggers the IFN effector pathways for creating the anti-viral state in host. PKR and the OAS)/RNAse L system is involved in the recognition and destruction of foreign RNA. Thus, avoidance of this system hinders the elicitation of viral RNA sensing as well as the virus-eliminating mechanisms by innate immunity	
Genetic mutations within SARS-CoV-2 spike (S) protein	Defective recognition or the inability of recognition of the receptor binding domain (RBD) of the viral spike (S) protein by the host angiotensin-converting enzyme 2 (ACE 2) receptor	Noor et al. (2022), Lazarevic et al. (2021), Korber et al. (2020), Zhang et al. (2020)

### Circumvention of innate immunity during viral entry

The nature of unusually large RNA genome of SARS-CoV-2 is of significance to the cellular innate immune sensors of the host that recognize these viruses upon their entry across the respiratory tract along with the successive downstream signaling cascades (like the IFN induced protective mechanisms) as expected to be induced (Zhao et al. 2022; Kikkert 2020). The ISGs as well as the IFN-induced transmembrane family (IFITM) proteins together with the lymphocyte antigen 6 complex locus E (*LY6E*) may interfere with the fusion across the host membrane (Kikkert 2020; Snijder et al. 2020). SARS-CoV-2 may employ several mechanisms which inhibit such IFN-I induction and signaling for the protection of the host (Kikkert 2020; Snijder et al. 2020; Klein et al. 2020). Patients with severe COVID-19 have been found with significantly impaired IFN-I signatures which unravels the IFN-mediated antiviral state evasion by the viral factors which are IFN antagonists as well as hinders the PRR sensing pathway used for protection of the host (Wan et al. 2020). Also, it has been already stated earlier that the spike (S) protein of SARS-CoV-2 mediates the viral entry into cells by binding to host cell surface receptor following fusion into the host membranes (Noor 2021a; Wan et al. 2020).

An interesting finding on this aspect is about the ADE-mediated viral entry whereby the neutralizing monoclonal antibody (MAb) targets the RBD of the spike protein; and allows it to undergo conformational changes making it prone to proteolytic activation (Fig. 1); and thus, mediates the viral entry into the IgG Fc receptor-expressing cells through the so-called canonical viral-receptor-dependent pathways (Kikkert 2020; Noor 2020; Wan et al. 2020). Surprisingly, while IFN seems to be the protective the early stage of the disease; however, along with the disease progression, the interferon-induced upregulation of ACE2 within the airway epithelia may contribute to the failure of IFN-1 induced antiviral state with a concomitant induction of other inflammatory pathways resulting in viral pathogenesis (Wan et al. 2020; Snijder et al. 2020).

### Modification of intracellular membranes for the ease of viral RNA replication

Viruses with unusually long positive RNA genome have been noticed to replicate exclusively in the cytosol by modifying intracellular membranes to form the so-called replication organelles (RO) or double membrane vesicles (DMVs) which may subsidize the evasion of the host innate immunity against the virus (Kikkert 2020; Snijder et al. 2020). Such specialized structure may have a rationale to spatially keep the viral RNA transcription and replication which would else be identified by the PRRs of the host innate immune protective sensors (Artika et al. 2020; Wang et al. 2020). Compared to the evidence-based data from the MERS-CoV and SARS-CoV-1, studies on such specialized structure for SARS-CoV-2 RNA replication are still insufficient. However, Klein and colleagues (in 2020) structurally characterized such curved membrane vesicles serving as the viral RNA replication compartment (or the budding site), and visualized the viral RNAs inside them (Klein et al. 2020).

### Safeguarding the viral RNA

It’s interesting to note that in order to make viral proteins, most viruses have been reported to hijack the host translational machineries, i.e., the host capping enzymes and utilizing the capped host mRNAs as substrates (Dai et al. 2020). The short, 5′-capped transcripts produced by the cellular DNA-dependent RNA polymerase II from the host mRNAs is the process that is known as the cap-snatching mechanism (Mandilara et al. 2021; Kikkert 2020). Such cap-snatching process has been observed in several positive stranded RNA viruses and in the Influenza virus which possess the viral mRNAs comprising both the host capped small RNA (addition of a 7-methyl guanosine; and lacking of the 2′-O-methylation) and the virus-encoded RNA (Dai et al. 2020; Drappier et al. 2015). SARS-CoV-2 RNA may employ several mechanisms to avoid the host immune response for its undisturbed transcription and translation. The viral RNAs may dodge recognition by the innate immune RNA sensors by adding a cap-structure to its 5′-end and hence the viral mRNAs can be appropriately recognized by the host translational machineries for the further translation (Kikkert 2020). However, the cap structure is not required for translation since these viral RNAs use a cap-independent internal ribosomal entry site-mediated translation rather it protects the viral RNA from the recognition by the innate RNA sensors (Kikkert 2020). Addition of 2'-O methylation to their cap-structures using nsp16 has been shown to be significant to avoid recognition by the MDA5 (melanoma differentiation-associated protein 5) sensor and consequent induction of the cells of innate immunity (Beyer and Forero 2022; Kikkert 2020).

Another mechanism of evasion of host innate immunity relies on the viral endoribonuclease activity encoded in one of the non-structural genes of the SARS-CoV-2 RNA (Kikkert 2020). This activity is essential for the avoidance of the MDA5, the protein kinase R (PKR), and the 2’-5’ Oligoadenylate Synthetase (the OAS/RNAse L system: one of the first characterized IFN effector pathways) machineries (Kikkert 2020; Drappier et al. 2015). PKR and the OAS)/RNAse L system recognize and destroy foreign RNA in the cytosol independently; and interestingly the virus seemingly razes its own RNA during certain stages of the infection to avoid eliciting the RNA sensing and virus-destroying immune system (Drappier et al. 2015; Kindler et al., 2017). This is also known that the cytosolic coronaviral mRNAs may be targeted by the cellular nonsense-mediated degradation pathway, resulting in the decay of these mRNAs (Kikkert 2020). However, the viral N protein has been reported to counteract such viral mRNA destruction possibly by packaging the viral RNAs (Kikkert 2020; Wada et al. 2018).

### Effectiveness of some vaccines against the SARS-CoV-2 variants: an emergency need

The Ad26.COV2.S (hAd26) vaccine (Janssen Vaccines & Prevention B.V.), also known as JNJ-78436735 has been found to be the most divergent vaccine against all sorts of S-mutations so far achieved as evaluated through the clinical trial NCT04505722 (Heinz and Stiasny 2021; Mercado et al. 2020; Coughlan 2020). The Ad26 vector encodes the prefusion stabilized S immunogen (S.PP) which consists of (1) the wild-type leader sequence, (2) the full-length membrane-bound spike protein, (3) mutant region of the furin cleavage site, and (4) two proline stabilizing mutation (Heinz and Stiasny 2021). The vaccine was found to elicit the production of the neutralizing antibodies which provided a complete protection against the viral challenge in animal model (Heinz and Stiasny 2021). Among the other vaccines, 86% record of vaccine efficiency was noted for the Sinovac Biotech (Sinovac, Beijing, China) vaccine; and the Sinopharm (Sinopharm, Beijing, China) whole-virus vaccines (Heinz and Stiasny 2021; Mercado et al. 2020; Forni et al. 2021; Wadman and Cohen 2021). Among the subunit vaccines, NVX-CoV2373 (Novavax, Gaithersburg, MD, USA) vaccine (a recombinant full-length S protein served as antigen with stabilizing mutations) was ended up with 90% efficiency in the Phase III clinical trials with an outcome of approximately 90% success in a trial in the UK; and 60% in South Africa (Wada et al. 2018, Wadman and Cohen 2021). The COVID-19 mRNA vaccines (from Biontech/Pfizer and Moderna) have already been being used in many countries after successful clinical trials with more than 94% efficiency which actually represents a massive dive forward for the COVID-19 mitigation worldwide (Noor 2021b; Heinz and Stiasny 2021).

This is to be noted that the UK mutant was found to be neutralized both by the Biontech/Pfizer- and the Moderna mRNA vaccine although a reduced neutralization of the South African variant by the Moderna vaccine was observed (Heinz and Stiasny 2021; Wu 2021). Likewise, the widely used Oxford/Astra Zeneca vaccine has also been found to be protective against the B.1.1.7 mutant; however, with a nine-fold lower efficiency which drives the scientists to think for the improvement of the vaccines against the SARS-CoV-2 variants (Emary et al. 2021; Heinz and Stiasny 2021). Such an incidence clearly urges the need for the improvement of the current vaccines in terms of their components, dosage forms, etc., or directs toward the need for multi-component/ combined vaccine or the seasonal vaccines as stated before (Noor 2021b). Based on a huge amount of literature recently published, Table 3 simply outlines the major COVID-19 vaccines which are currently in use around the world. One important is to ponder that since the SARS-CoV-2 and its variants may escape the host immunity, substantial non-pharmaceutical interventions are required to limit the rate of SARS-CoV-2 transmission within a community or in a country (Bhuiyan et al. 2022; Noor 2022b; Noor et al. 2022; Perra 2021).

**Table 3:** Available major COVID-19 vaccines (Phase III) against SARS-CoV-2 and its variants

Major vaccines	Composition of vaccine	Mode of action against SARS-CoV-2 and its variants	References
BioNTech/ Pfizer mRNA vaccine BNT162: a1, b1, b2, c2	Lipid nano-particle (LNP)-encapsulated mRNA vaccine encoding spike (S) protein	Target: Spike (S) protein with two stabilizing proline mutations within the S2 subunit Possible target: Spike (S) protein	Noor (2021a, b b, 2021c), Ura et al. (2021)
Moderna mRNA-1273 vaccine			
AstraZeneca/ University of Oxford ChAdOx1 nCoV-19**/**AZD1222 replicating vector vaccine	Attenuated version of a common cold virus (adenovirus): the genetic material has been added to the ChAdOx1 construct	Target: Spike (S) protein. High doses of vector particles are administered followed by recognition by the host immune sensors; resulting in the induction of pro-inflammatory cytokines and chemokines	Noor (2021a, b b, 2021c), Ura et al. (2021), Mercado et al. (2020)
Johnson & Johnson Ad26 vector-based vaccine: Ad26.COV2.S	Adenovirus-type 26 non-replicating viral vector which expresses the S protein. Seven (7) variants of the SARS-CoV-2 spike (S) protein sequences are codon optimized and artificially synthesized. So far the most divergent vaccine with S protein variants as target	Activates S-specific and RBD-specific neutralizing antibody production; (2) triggers cell-mediated immunity. Ad26 vector encodes the prefusion stabilized S immunogen (wild-type leader sequence, full-length membrane-bound S, mutation of the furin cleavage site, and two proline stabilizing mutation)	Noor (2021a, b b, 2021c), Ura et al. (2021), Mercado et al. (2020)
Gam-COVID-Vac/ Sputnik V two vector COVID-19 vaccine	Mixture of the recombinant replication-defective adenovirus serotype 26 (Ad 26) plus Ad5		
Sinovac and Sinopharm BBIBP-CorV vaccine	Vero cell grown vaccine, inactivated by β-propiolactone (BPL) and the vaccine immunogenicity is increased by the addition of adjuvants	Production of S protein neutralizing antibodies; elicitation of cell-mediated immunity	Noor et al. (2022), Heinz and Stiasny (2021), Gao et al. (2020)

## Conclusions

The endurance of the ongoing wave of the COVID-19 pandemic is quite likely to generate the immunologically pertinent mutations within the SARS-CoV-2 which may be resistant against the currently used vaccines. The strategy of escaping the host protective immunity by SARS-CoV-2 with the concomitant production of the mutant/ variants, i.e., mutations in the viral spike (S) protein have made the virus incrementally lethal to mass public. Therefore, the possible impact of the viral variants on the instigation of the appropriate immunity provoked by the vaccines which are currently being used needs to be carefully assessed. Besides, rigorous surveillance of the emerging variants through genome sequencing based on the geographical locations, study of the transmission dynamics, and both the pharmaceutical and non-pharmaceutical interventions (like isolation/ quarantine/ lockdown) are required. Indeed, at present the COVID-19 vaccine booster doses are highly suggested as the VOCs may possess the capacity of escaping as well as counteracting the neutralizing antibodies together with the cell-mediated protections host immune system.

## Data Availability

Not applicable.

## Abbreviations

ACE-2: Angiotensin-converting enzyme 2
ADE: Antibody-dependent entry
ARDS: Acute respiratory distress syndrome
ASRD: Aspirin-sensitive respiratory disease
DCs: Dendritic cells
G-CSF: Granulocyte colony-stimulating factor
GISAID: Global initiative for sharing all influenza data
GM-CSF: Granulocyte-macrophage colony-stimulating factor
IFN: Interferon
IL 1RA: Interleukin-1 receptor antagonist
ILs: Interleukins
IP 10: IFNγ-induced protein 10
MCP1: Monocyte chemotactic protein-1
MDA5: Melanoma differentiation-associated protein 5
MERS-CoV: Middle east respiratory syndrome coronavirus
MIP1α: Macrophage inflammatory protein 1 α
Nsps: Non-structural proteins
ORFs: Open reading frames
PAMPs: Pathogen-associated molecular patterns
PDGF: Platelet-derived growth factor
PRRs: Pattern recognition receptors
RBD: Receptor binding domain
RdRp: RNA-dependent RNA polymerase
RTC: Replicase–transcriptase complex
S. PP: Prefusion-stabilized S immunogen
SARS-CoV-2: Severe acute respiratory coronavirus 2
TLRs: Toll-like receptors
TMPRSS2: Transmembrane protease serine 2
TNF: Tumor necrosis factor
VEGFA: Vascular endothelial growth factor A
VLPs: Virus like particles
VOCs: Variants of concern
VOIs: Variants of interest

## References

[CR1] Abdelghany TM, Ganash M, Bakri MM, Qanash H, Al-Rajhi AMH, Elhussieny NI (2021). SARS-CoV-2, the other face to SARS-CoV and MERS-CoV: future predictions. Biomed J.

[CR2] Artika IM, Dewantari AK, Wiyatno A (2020). Molecular biology of coronaviruses: current knowledge. Heliyon.

[CR3] Awadasseid A, Wu Y, Tanaka Y, Zhang W (2021). SARS-CoV-2 variants evolved during the early stage of the pandemic and effects of mutations on adaptation in Wuhan populations. Int J Biol Sci.

[CR4] Beyer DK, Forero A (2022). Mechanisms of antiviral immune evasion of SARS-CoV-2. J Mol Biol.

[CR5] Bhat EA, Khan J, Sajjad N, Ali A, Aldakeel FM, Mateen A, Alqahtani MS, Syed R (2021). SARS-CoV-2: insight in genome structure, pathogenesis and viral receptor binding analysis-an updated review. Int Immunopharmacol.

[CR6] Bhuiyan AA, Brahmachari S, Ripa IJ, Noor R (2022). Overview of dreadful consequences of SARS-CoV-2 invasion in Italy from March 2020 to March 2022. Bull Natl Res Cent.

[CR7] Birhanu A, Ayana GM, Merga BT, Alemu A, Negash B, Seid A, Dessie Y (2022). Incidence and predictors of organ failure among COVID-19 hospitalized adult patients in Eastern Ethiopia. Hospital-based retrospective cohort study. BMC Infect Dis.

[CR8] Boehm E, Kronig I, Neher RA, Eckerle I, Vetter P, Kaiser L (2021). Geneva centre for emerging viral diseases. Novel SARS-CoV-2 variants: the pandemics within the pandemic. Clin Microbiol Infect.

[CR9] Bonnet B, Cosme J, Dupuis C, Coupez E, Adda M, Calvet L, Fabre L, Saint-Sardos P, Bereiziat M, Vidal M, Laurichesse H, Souweine B, Evrard B (2021). Severe COVID-19 is characterized by the co-occurrence of moderate cytokine inflammation and severe monocyte dysregulation. EBioMedicine.

[CR10] Cacciapaglia G, Cot C, Sannino F (2020). Second wave COVID-19 pandemics in Europe: a temporal playbook. Sci Rep.

[CR11] Campbell F, Archer B, Laurenson-Schafer H, Jinnai Y, Konings F, Batra N, Pavlin B, Vandemaele K, Van Kerkhove MD, Jombart T, Morgan O, de Le Polain WO (2021). Increased transmissibility and global spread of SARS-CoV-2 variants of concern as at June 2021. Euro Surveill.

[CR12] Chenchula S, Karunakaran P, Sharma S, Chavan M (2022). Current evidence on efficacy of COVID-19 booster dose vaccination against the Omicron variant: a systematic review. J Med Virol.

[CR13] Coughlan L (2020). Factors which contribute to the immunogenicity of non-replicating adenoviral vectored vaccines. Front Immunol.

[CR14] Dai H, Gu W (2020). Small RNA plays important roles in virus-host interactions. Viruses.

[CR15] Dos Santos WG (2021). Impact of virus genetic variability and host immunity for the success of COVID-19 vaccines. Biomed Pharmacother.

[CR16] Drappier M, Michiels T (2015). Inhibition of the OAS/RNase L pathway by viruses. Curr Opin Virol.

[CR17] Emary KRW, Golubchik T, Aley PK, Ariani CV, Angus B, Bibi S (2021). Efficacy of ChAdOx1 nCoV-19 (AZD1222) vaccine against SARS-CoV-2 variant of concern 202012/01 (B.1.1.7): an exploratory analysis of a randomised controlled trial. Lancet.

[CR18] Forni G, Mantovani A (2021). COVID-19 Commission of Accademia Nazionale dei Lincei, Rome. COVID-19 vaccines: where we stand and challenges ahead. Cell Death Differ.

[CR19] Gao Q, Bao L, Mao H, Wang L, Xu K, Yang M (2020). Development of an inactivated vaccine candidate for SARS-CoV-2. Science.

[CR20] Gu W, Gan H, Ma Y, Xu L, Cheng ZJ, Li B, Zhang X, Jiang W, Sun J, Sun B, Hao C (2022). The molecular mechanism of SARS-CoV-2 evading host antiviral innate immunity. Virol J.

[CR21] Han P, Li L, Liu S, Wang Q, Zhang D, Xu Z (2022). Receptor binding and complex structures of human ACE2 to spike RBD from omicron and delta SARS-CoV-2. Cell.

[CR22] Haider N, Yavlinsky A, Simons D, Osman AY, Ntoumi F, Zumla A, Kock R (2020). Passengers' destinations from China: low risk of Novel Coronavirus (2019-nCoV) transmission into Africa and South America. Epidemiol Infect.

[CR23] Heinz FX, Stiasny K (2021). Profiles of current COVID-19 vaccines. Wien Klin Wochenschr.

[CR24] Hembram P (2021). An outline of SARS-CoV-2 pathogenesis and the complement cascade of immune system. Bull Natl Res Cent.

[CR25] Hoffmann M, Hofmann-Winkler H, Krüger N, Kempf A, Nehlmeier I, Graichen L. et al. (2021) SARS-CoV-2 variant B.1.617 is resistant to Bamlanivimab and evades antibodies induced by infection and vaccination. bioRxiv preprint. 10.1101/2021.05.04.442663 10.1016/j.celrep.2021.109415 PMC8238662 34270919

[CR26] Jackson CB, Farzan M, Chen B, Choe H (2022). Mechanisms of SARS-CoV-2 entry into cells. Nat Rev Mol Cell Biol.

[CR27] Kaushal A, Noor R (2022). Association of gut microbiota with inflammatory bowel disease and COVID-19 severity: a possible outcome of the altered immune response. Curr Microbiol.

[CR28] Khare S, Gurry C, Freitas L, Schultz MB, Bach G, Diallo A, Akite N, Ho J, Lee RT, Yeo W (2021). Curation Team GC, Maurer-Stroh S. GISAID's Role in Pandemic Response. China CDC Wkly..

[CR29] Kikkert M (2020). Innate immune evasion by human respiratory RNA viruses. J Innate Immun.

[CR30] Kindler E, Gil-Cruz C, Spanier J, Li Y, Wilhelm J, Rabouw HH (2017). Early endonuclease-mediated evasion of RNA sensing ensures efficient coronavirus replication. PLoS Pathog.

[CR31] Klein S, Cortese M, Winter SL, Wachsmuth-Melm M, Neufeldt CJ, Cerikan B (2020). SARS-CoV-2 structure and replication characterized by in situ cryo-electron tomography. Nat Commun.

[CR32] Korber B (2020). Tracking changes in SARS-CoV-2 spike: evidence that D614G increases infectivity of the COVID-19 virus. Cell.

[CR33] Kumar A, Parashar R, Kumar S, Faiq MA, Kumari C, Kulandhasamy M, Narayan RK, Jha RK, Singh HN, Prasoon P, Pandey SN, Kant K (2022). Emerging SARS-CoV-2 variants can potentially break set epidemiological barriers in COVID-19. J Med Virol.

[CR34] Lazarevic I, Pravica V, Miljanovic D, Cupic M (2021). Immune evasion of SARS-CoV-2 emerging variants: what have we learnt so far?. Viruses.

[CR35] Li G, Fan Y, Lai Y, Han T, Li Z, Zhou P (2020). Coronavirus infections and immune responses. J Med Virol.

[CR36] Madden EA, Diamond MS (2022). Host cell-intrinsic innate immune recognition of SARS-CoV-2. Curr Opin Virol.

[CR37] Mattoo SU, Kim SJ, Ahn DG, Myoung J (2022). Escape and over-activation of innate immune responses by SARS-CoV-2: two faces of a coin. Viruses.

[CR38] Mandilara G, Koutsi MA, Agelopoulos M, Sourvinos G, Beloukas A, Rampias T (2021). The role of coronavirus RNA-processing enzymes in innate immune evasion. Life (Basel).

[CR39] Martin-Sancho L, Lewinski MK, Pache L, Stoneham CA, Yin X, Becker ME, Pratt D, Churas C, Rosenthal SB, Liu S, Weston S, De Jesus PD, O'Neill AM, Gounder AP, Nguyen C, Pu Y, Curry HM, Oom AL, Miorin L, Rodriguez-Frandsen A, Zheng F, Wu C, Xiong Y, Urbanowski M, Shaw ML, Chang MW, Benner C, Hope TJ, Frieman MB, García-Sastre A, Ideker T, Hultquist JF, Guatelli J, Chanda SK (2021). Functional landscape of SARS-CoV-2 cellular restriction. Mol Cell.

[CR40] Mercado NB, Zahn R, Wegmann F, Loos C, Chandrashekar A, Yu J (2020). Single-shot Ad26 vaccine protects against SARS-CoV-2 in rhesus macaques. Nature.

[CR41] Naqvi AAT, Fatima K, Mohammad T, Fatima U, Singh IK, Singh A (2020). Insights into SARS-CoV-2 genome, structure, evolution, pathogenesis and therapies: structural genomics approach. Biochim Biophys Acta Mol Basis Dis.

[CR42] Noor R, Maniha SM (2020). A brief outline of respiratory viral disease outbreaks: 1889-till date on the public health perspectives. VirusDis.

[CR43] Noor R (2020). Antiviral drugs against severe acute respiratory syndrome coronavirus 2 infection triggering the coronavirus disease-19 pandemic. Tzu Chi Med J.

[CR44] Noor R (2021). A comparative review of pathogenesis and host innate immunity evasion strategies among the severe acute respiratory syndrome coronavirus 2 (SARS-CoV-2), severe acute respiratory syndrome coronavirus (SARS-CoV) and the Middle East respiratory syndrome coronavirus (MERS-CoV). Arch Microbiol.

[CR45] Noor R (2021). Developmental status of the potential vaccines for the mitigation of the COVID-19 pandemic and a focus on the effectiveness of the Pfizer-BioNTech and moderna mRNA vaccines. Curr Clin Microbiol Rep.

[CR46] Noor R (2021). A review on the effectivity of the current COVID-19 drugs and vaccines: are they really working against the severe acute respiratory syndrome coronavirus 2 (SARS-CoV-2) variants?. Curr Clin Micro Rpt.

[CR47] Noor R, Shareen S, Billah M (2022). COVID-19 vaccines: their effectiveness against the emerging severe acute respiratory syndrome coronavirus 2 (SARS-CoV-2) and its emerging variants. Bull Natl Res Cent.

[CR48] Noor R (2022). A review on the induction of host immunity by the current COVID-19 vaccines and a brief non-pharmaceutical intervention to mitigate the pandemic. Bull Natl Res Cent.

[CR49] Noor R (2022). mRNA vaccines as an efficient approach for the rapid and robust induction of host immunity against SARS-CoV-2. SN Compr Clin Med.

[CR50] Otto SP, Day T, Arino J, Colijn C, Dushoff J, Li M, Mechai S, Van Domselaar G, Wu J, Earn DJD, Ogden NH (2021). The origins and potential future of SARS-CoV-2 variants of concern in the evolving COVID-19 pandemic. Curr Biol.

[CR51] Perra N (2021). Non-pharmaceutical interventions during the COVID-19 pandemic: a review. Phys Rep.

[CR52] Raj R (2020). Analysis of non-structural proteins, NSPs of SARS-CoV-2 as targets for computational drug designing. Biochem Biophys Rep.

[CR53] Redondo N, Zaldívar-López S, Garrido JJ, Montoya M (2021). SARS-CoV-2 accessory proteins in viral pathogenesis: knowns and unknowns. Front Immunol.

[CR54] Romano M, Ruggiero A, Squeglia F, Maga G, Berisio R (2020). A structural view of SARS-CoV-2 RNA replication machinery: RNA Synthesis, proofreading and final capping. Cells.

[CR55] Sanyaolu A, Marinkovic A, Prakash S, Haider N, Williams M, Okorie C, Badaru O, Smith S (2022). SARS-CoV-2 omicron variant (B.1.1.529): a concern with immune escape. World J Virol.

[CR56] Samantaray A, Johnson E, Kumar N, Mehdiratta L (2021). COVID-19: a game of drugs, vaccines, hope and… death!. Indian J Anaesth.

[CR57] Snijder EJ, Limpens RWAL, de Wilde AH, de Jong AWM, Zevenhoven-Dobbe JC, Maier HJ (2020). A unifying structural and functional model of the coronavirus replication organelle: tracking down RNA synthesis. PLoS Biol.

[CR58] Taefehshokr N, Taefehshokr S, Hemmat N, Heit B (2020). Covid-19: perspectives on innate immune evasion. Front Immunol.

[CR59] Trougakos IP, Stamatelopoulos K, Terpos E, Tsitsilonis OE, Aivalioti E, Paraskevis D, Kastritis E, Pavlakis GN, Dimopoulos MA (2021). Insights to SARS-CoV-2 life cycle, pathophysiology, and rationalized treatments that target COVID-19 clinical complications. J Biomed Sci.

[CR60] Tian F, Tong B, Sun L, Shi S, Zheng B, Wang Z, Dong X, Zheng P (2021). N501Y mutation of spike protein in SARS-CoV-2 strengthens its binding to receptor ACE2. Elife.

[CR61] Ura T, Yamashita A, Mizuki N, Okuda K, Shimada M (2021). New vaccine production platforms used in developing SARS-CoV-2 vaccine candidates. Vaccine.

[CR62] Vabret N, Britton GJ, Gruber C, Hegde S, Kim J, Kuksin M (2020). Immunology of COVID-19: current state of the science. Immunity.

[CR63] van der Made CI, Simons A, Schuurs-Hoeijmakers J, van den Heuvel G, Mantere T, Kersten S, van Deuren RC, Steehouwer M, van Reijmersdal SV, Jaeger M, Hofste T, Astuti G, Corominas Galbany J, van der Schoot V, van der Hoeven H, Hagmolen W, Klijn E, van den Meer C, Fiddelaers J, de Mast Q, Bleeker-Rovers CP, Joosten LAB, Yntema HG, Gilissen C, Nelen M, van der Meer JWM, Brunner HG, Netea MG, van de Veerdonk FL, Hoischen A (2020). Presence of genetic variants among young men with severe COVID-19. JAMA.

[CR64] V’kovski P, Kratzel A, Steiner S, Stalder H, Thiel V (2021). Coronavirus biology and replication: implications for SARS-CoV-2. Nat Rev Microbiol.

[CR65] Veerabathiran R, Ragunath B, Kaviarasan V, Mohammed V, Ahmed SSSJ (2021). Identification of selected genes associated with the SARS-CoV-2: a therapeutic approach and disease severity. Bull Natl Res Cent.

[CR66] Wada M, Lokugamage KG, Nakagawa K, Narayanan K, Makino S (2018). Interplay between coronavirus, a cytoplasmic RNA virus, and nonsense-mediated mRNA decay pathway. Proc Natl Acad Sci U S A.

[CR67] Wadman M, Cohen J (2021). Novavax vaccine delivers 89% efficacy against COVID-19 in UK—but is less potent in South Africa. Science.

[CR68] Wan Y, Shang J, Sun S, Tai W, Chen J, Geng Q (2020). Molecular mechanism for antibody-dependent enhancement of coronavirus entry. J Virol.

[CR69] Wang Y, Grunewald M, Perlman S (2020). Coronaviruses: an updated overview of their replication and pathogenesis. Methods Mol Biol.

[CR70] WHO (World Health Organization) (2022) Coronavirus diseases (COVID-19) Dashboard. Updated on 4:49pm CEST, 27 June 2022. https://covid19.who.int/ Accessed 28 June 2022

[CR71] Wrapp D, Wang N, Corbett KS, Goldsmith JA, Hsieh CL, Abiona O (2020). Cryo-EM structure of the 2019-nCoV spike in the prefusion conformation. Science.

[CR72] Wu K, Werner AP, Moliva JI, Koch M, Choi A, Stewart-Jones GBE (2021). mRNA-1273 vaccine induces neutralizing antibodies against spike mutants from global SARS-CoV-2 variants. bioRxiv.

[CR73] Yin X, Riva L, Pu Y, Martin-Sancho L, Kanamune J, Yamamoto Y, Sakai K, Gotoh S, Miorin L, De Jesus PD, Yang CC, Herbert KM, Yoh S, Hultquist JF, García-Sastre A, Chanda SK (2021). MDA5 governs the innate immune response to SARS-CoV-2 in lung Epithelial cells. Cell Rep.

[CR74] Zhang L, Jackson CB, Mou H, Ojha A, Rangarajan ES, Izard T, Farzan M, Choe H (2020). The D614G mutation in the SARS-CoV-2 spike protein reduces S1 shedding and increases infectivity. bioRxiv.

[CR75] Zhao X, Chen D, Li X, Griffith L, Chang J, An P, Guo JT (2022). Interferon control of human coronavirus infection and viral evasion: mechanistic insights and implications for antiviral drug and vaccine development. J Mol Biol.

